# Radiomics-based machine learning model for predicting clinically ineffective reperfusion in acute ischaemic stroke patients after endovascular treatment

**DOI:** 10.3389/fneur.2025.1606287

**Published:** 2025-09-18

**Authors:** Xiaolong Hu, Suya Li, Shifei Ye, Zhiliang Ding, Peng Li, Yibin Fang

**Affiliations:** ^1^Tongji University Affiliated Shanghai 4th People’s Hospital, Tongji University School of Medicine, Shanghai, China; ^2^Department of Neurovascular Disease, Tongji University Affiliated Shanghai 4th People’s Hospital, Shanghai, China; ^3^Nanjing Medical University Affiliated Suzhou Municipal Hospital, Nanjing, China

**Keywords:** thrombus, radiomics, machine learning, clinically ineffective reperfusion, stroke

## Abstract

**Background:**

Patients with acute ischaemic stroke (AIS) undergoing endovascular treatment may have a poor prognosis, even with successful recanalization. This study aims to evaluate a machine learning model based on CT-thrombosis radiomics to assess clinically ineffective reperfusion (CIR) after endovascular treatment (EVT) in patients with AIS.

**Methods:**

A total of 144 patients from two centres were included in this study, spanning from December 2021 to October 2024. The participants were randomly divided into a training set (70%) and a test set (30%). Patient outcomes were defined as clinically ineffective reperfusion (thrombolysis in cerebral infarction, TICI ≥2b, three-month post-surgery modified Rankin Scale, mRS ≥3) and effective reperfusion (TICI ≥2b, three-month post-surgery mRS <3). A total of 1,702 features were extracted from the intrathrombus and perithrombus regions. The minimum redundancy maximum relevance (mRMR) and least absolute shrinkage and selection operator (LASSO) algorithm were used for feature selection to construct the machine learning model, with the AUC of the receiver operating characteristic (ROC) curve used for model evaluation.

**Results:**

In the test set, the random forest (RF) model demonstrated the highest diagnostic performance among all the models (RF_INTRA AUC = 0.78, RF_PERI AUC = 0.76, RF_F AUC = 0.83).

**Conclusion:**

The machine learning model based on intrathrombus and perithrombus radiomics features can accurately predict clinically ineffective reperfusion in patients after EVT. However, further study is needed to validate these findings in larger, independent cohorts and explore the broader clinical applicability of the model.

## Introduction

Acute ischaemic stroke (AIS) is a cerebrovascular disease associated with a high burden of disability and mortality (1, 2). Endovascular therapy (EVT) is an established intervention for managing acute large vessel occlusion (3, 4). However, EVT is associated with complications, such as post-thrombectomy bleeding and cerebral oedema caused by ischaemia-reperfusion (5, 6). Therefore, not all patients with AIS benefit from EVT. Consequently, it is crucial to find effective methods of identifying patients suitable for EVT and accurately predicting successful reperfusion.

Traditional prognostic imaging markers, such as hyper-dense middle cerebral artery signs (7), large core infarcts (8), and CT perfusion mismatch (9), are considered indicators of patient prognosis. However, these existing imaging biomarkers rely on subjective interpretation, resulting in varying conclusions among clinicians, limiting their reliability in predicting successful reperfusion. Therefore, an objective and accurate approach to predicting successful reperfusion is urgently needed.

Radiomics (10), is a quantitative imaging analysis technique designed to facilitate the extraction of numerous quantitative features from medical images. These features aid in disease diagnosis, treatment outcome prediction, and assessment of therapeutic efficacy.

Machine learning complements radiomics by efficiently processing large datasets, automatically optimising the model performance, and identifying complex patterns. Advanced models such as support vector machine (SVM) and K-nearest neighbors (KNN) often outperform traditional statistical models in terms of predictive efficacy (11). Current research on thrombus radiomics has primarily focused on thrombus composition (12), identification of cardiovascular stroke (13), and prediction of the first-pass effect (14) and has yielded notable promising results. However, a gap persists in leveraging this approach for predicting effective recanalization.

In this study, we developed a machine learning model based on the radiomics features derived from the thrombus and surrounding tissue to accurately predict whether CIR occurs after EVT in patients with AIS. Our goal was to facilitate the identification of patients with AIS who are unsuitable for EVT and to provide informed guidance for future clinical practice.

## Materials and methods

### Patients

We conducted a retrospective analysis of all patients with AIS admitted to the two participating hospital stroke centres between December 2021 and October 2024 (Centre A, *n* = 109; Centre B, *n* = 35). The inclusion criteria were as follows: (1) acute ischaemic stroke caused by large-vessel occlusion; (2) visible thrombosis on CT; (3) undergoing EVT; and (4) availability of complete clinical and imaging data. Exclusion criteria included: (1) incomplete imaging data or low-quality images unsuitable for radiomic feature extraction; and (2) loss to follow-up at 3 months post-surgery. CIR was defined as achieving a TICI score of ≥2b post-thrombectomy and a modified Rankin Scale (mRS) score ≥3 at three-month follow-up. This study followed the Helsinki guidelines and received approval from the institutional ethics review board. Informed consent was obtained from all patients.

### Image acquisition and thrombus segmentation

**Figure 1:**
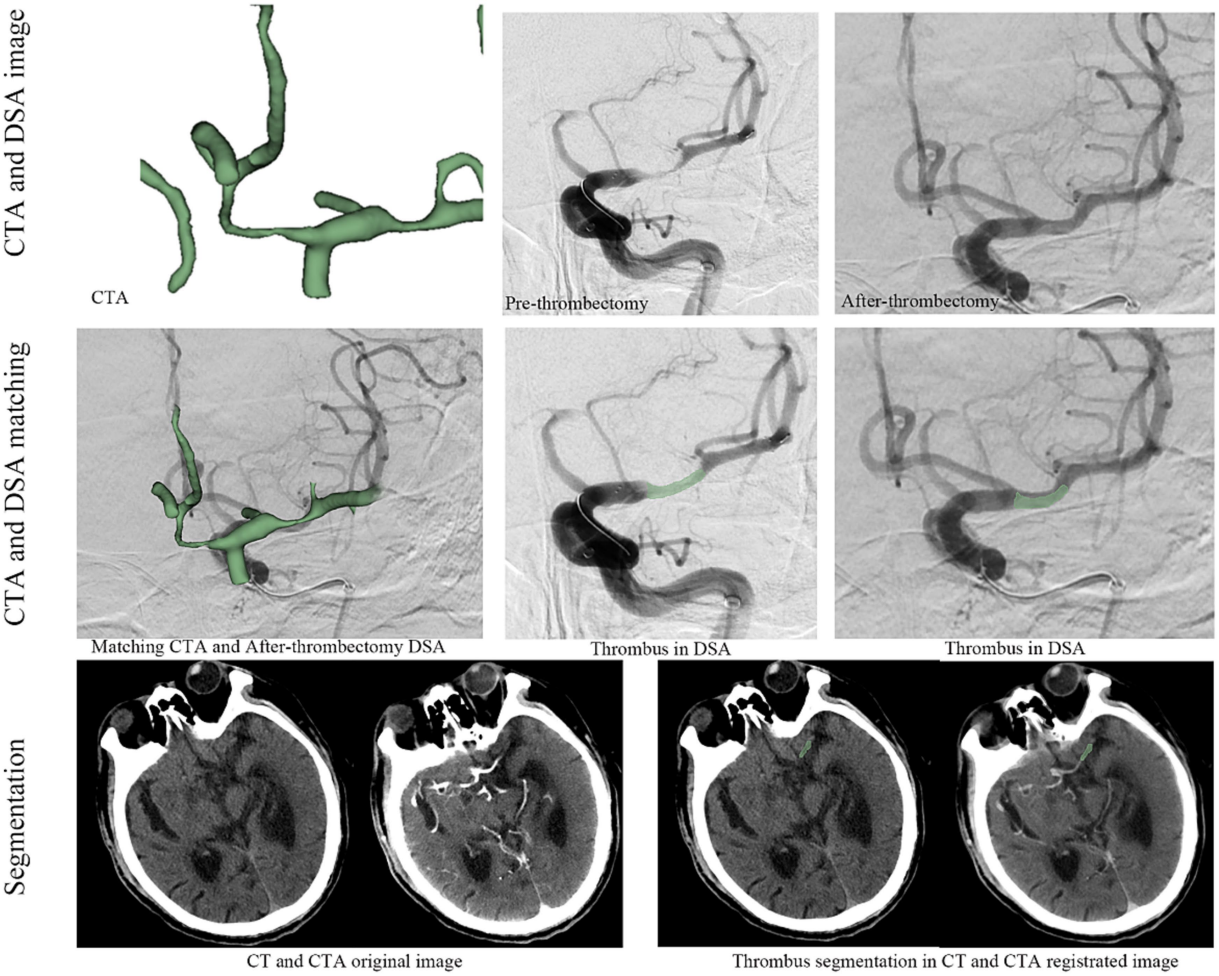
The workflow for thrombus radiomics feature extraction.

CT and CTA images were obtained using 64-slice, 128-row CT scans (Siemens, Germany) with a slice thickness of 0.5 mm. Preoperative CTA images were matched to postoperative DSA images using ANTS software to accurately identify the thrombus (Figure 1). Thrombus segmentation was performed based on the matching results of CTA and postoperative DSA; the location of the thrombus was annotated using NCCT and CTA (Figure 1). Segmentation was conducted by two experienced clinicians using 3D Slicer (version 4.9.0, National Institutes of Health) with verification by another senior physician. Appropriate measures were taken to minimise patient discomfort during the procedures. Following thrombus segmentation, Python (version 2.7.13) was used to automatically segment the perithrombus areas within a 1 mm boundary. Additionally, researchers were blinded to the patients’ clinical information.

### Radiomics parameters (RFs) extraction and selection

**Figure 2:**
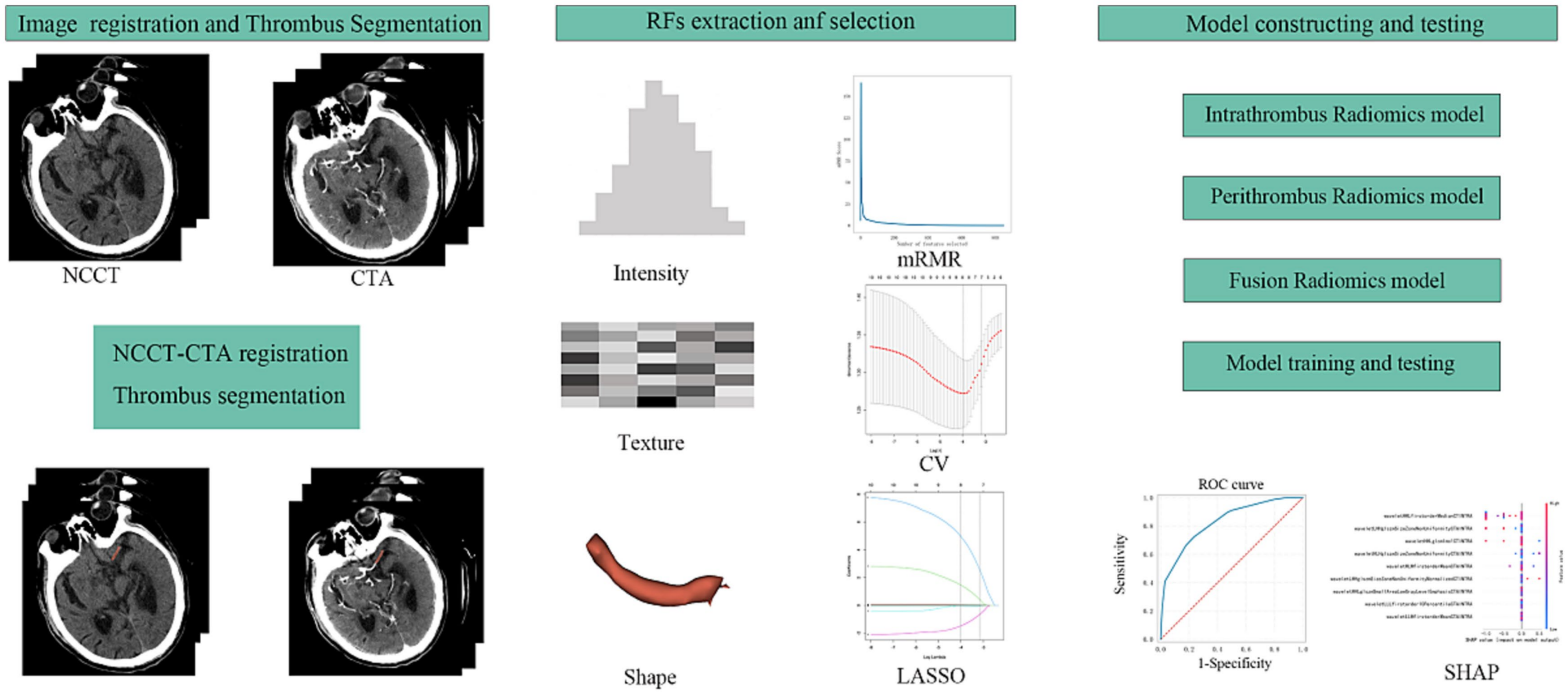
The workflow of this study design. RFs, radiomics features; mRMR, maximal relevance and minimal redundancy; CV, cross validation; LASSO, least absolute shrinkage selection operator; SHAP, SHapley Additive exPlanations.

All images were resampled to a voxel size of 1 mm × 1 mm × 1 mm, and radiomics parameters were extracted using radiomics. Thrombus segmentation and radiomics parameter extraction were conducted by two experienced clinicians. Data consistency was evaluated using the intraclass correlation coefficient (ICC), with parameters having an ICC >0.8 included in subsequent analyses. *Z*-scores were used to standardise the data across the entire dataset, and the minimum redundancy maximum relevance (mRMR) algorithm (Figure 2) was used to select the top 10 variables for further study. The least absolute shrinkage and selection operator (LASSO) was applied to refine the selection of variables for subsequent model construction.

### Model building and evaluation

Datasets were randomly divided into training and testing sets in a 7:3 ratio. A 10-fold cross-validation was used applied to the training set for model development. Diagnostic performance was evaluated on the testing set using receiver operating characteristic (ROC) curves (Figure 2). Machine learning models were constructed for the intrathrombus and perithrombus areas, labelled RF_INTRA, DT_INTRA, SVM_INTRA, and KNN_INTRA for intrathrombus, and RF_PERI, DT_PERI, SVM_PERI, and KNN_PER for perithrombus. Fusion models (RF_F, DT_F, SVM_F, and KNN_F) were developed by integrating features from both regions.

We selected the point on the ROC curve with the minimum distance from the left-upper corner of the curve and used the corresponding sensitivities and specificities to calculate likelihood ratios. LR^+^ represents the ratio of the probability of a positive test result in patients with the condition to the probability in those without the condition, while LR^−^ reflects the opposite. The formulas were as follows: LR^+^ = Sensitivity/(1 − Specificity), LR^−^ = (1 − Sensitivity)/Specificity.

The clinical impact of LRs was interpreted using established thresholds: LR^+^ >10 or LR^−^ <0.1 indicate strong evidence to rule in or rule out a diagnosis, respectively. LR^+^ 5–10 or LR^−^ 0.1–0.2 suggest moderate diagnostic value. LR^+^ 2–5 or LR^−^ 0.2–0.5 provide limited but potentially useful shifts in probability.

### SHAP analysis

**Figure 3:**
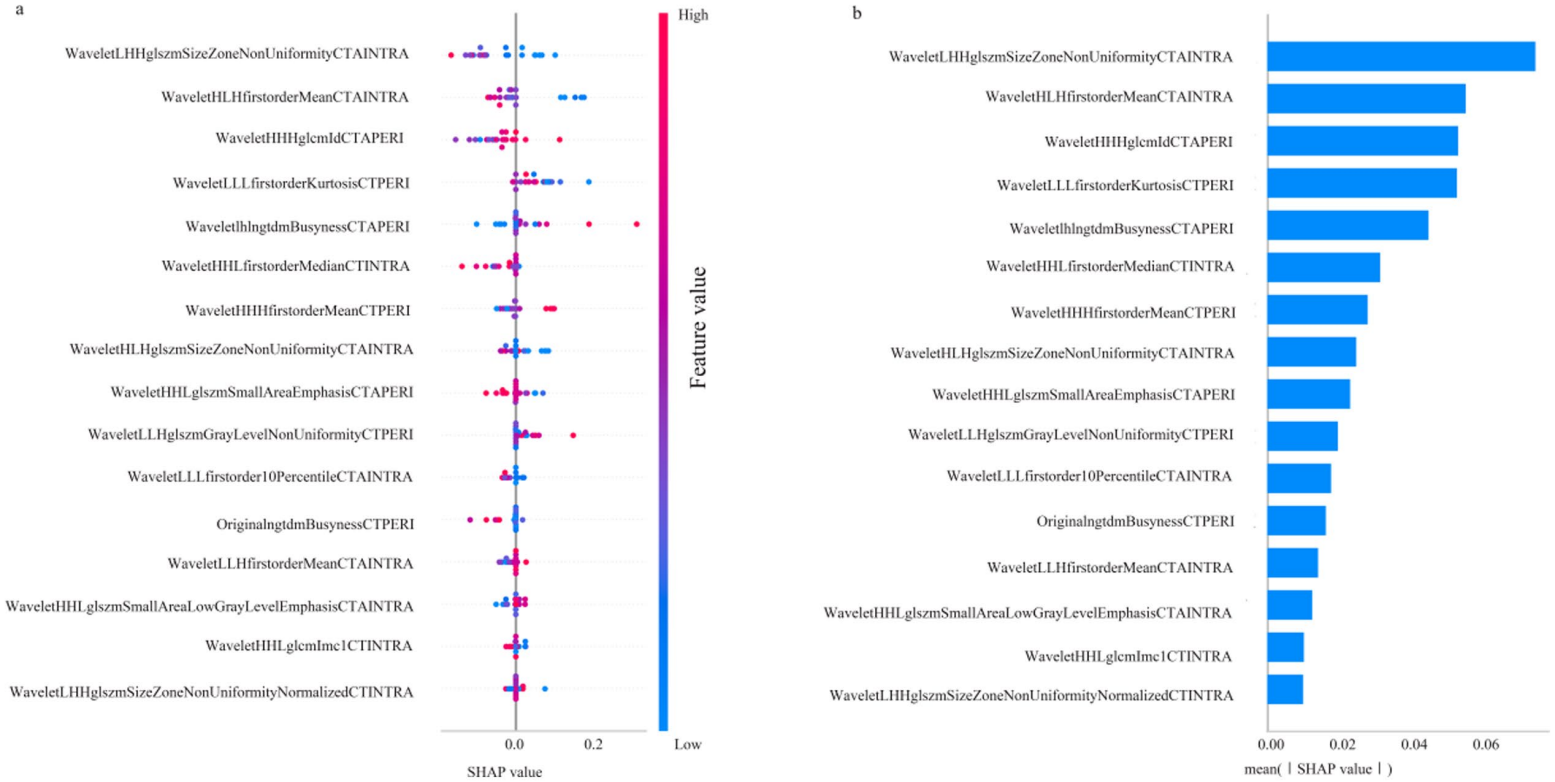
SHAP diagram of RF_F model. **(a)** SHAP honeycomb diagram of RF_F model. **(b)** SHAP value ranking of the variables in the RF_F model.

The SHAP algorithm was used to assess the contribution of each parameter in the RF-F model. The variable importance plot ranked the impact of each parameter on the model performance according to the SHAP values (Figure 3).

### Statistical analysis

Statistical analyses were conducted using R software (version 4.2.2), primarily using the glmnet, e1071, and caTools packages. Quantitative data following normal distribution were reported as mean ± standard deviation (M ± SD), and group differences were compared using the *t*-test. Non-normally distributed quantitative data were reported as the M, Q1, and Q3, and group comparisons were conducted using the Mann–Whitney *U* test. Qualitative data were presented as frequencies (*N*) and percentages (%), with group differences assessed using the chi-square test or Fisher’s exact test for sample sizes <5. Statistical significance was set at *p* < 0.05. The Test value refers to the test statistic (e.g., *t*-value for *t*-tests or chi-square value for chi-square tests) generated during hypothesis testing. It quantifies the difference or association between groups.

## Results

### Patients characteristics

A total of 144 patients were included in this study, of whom 45 (31.25%) experienced clinically ineffective reperfusion after EVT. Table 1 presents the baseline characteristics of the effective and ineffective reperfusion groups. No significant differences were observed between the groups regarding age, sex, hypertension, diabetes, or NIHSS scores (*p* > 0.05).

**Table 1:** Patients’ baseline characteristics.

Characteristics	Effective reperfusion (*n* = 99)	Clinically ineffective reperfusion (*n* = 45)	Test value	*p*-value
Age	71 ± 13	76 ± 11	2.14	0.97
Sex			0.09	0.77
Male	51	22		
Female	48	23		
Hypertension	50	28	1.71	0.19
Diabetes	34	15	0.014	0.91
NIHSS	10 ± 9	17 ± 9	4.21	0.29
Site				
ICA	23	12	—	>0.05
MCA	60	24	—	>0.05
M1	43	17	—	—
M2	12	6	—	—
M3	5	1	—	—
ACA	1	0	—	>0.05
VA	11	6	—	>0.05
BA	1	3	—	>0.05
PCA	3	0	—	>0.05

### Feature extraction and selection

From the intrathrombus and perithrombus area, 1,702 features were extracted, including 28 shape features, 36 first-order features, 150 texture features, and 1,488 wavelet features. All parameters had an ICC greater than 0.8, ensuring their inclusion in subsequent analyses. The final variables used to develop the model were selected using the mRMR and LASSO algorithms, including:

waveletLHHglszmSizeZoneNonUniformityCTAINTRA, waveletHLHfirstorderMeanCTAINTRA, waveletHHLfirstorderMedianCTINTRA, waveletHHLglszmSmallAreaLowGrayLevelEmphasisCTAINTRA, waveletLLLfirstorder10PercentileCTAINTRA, waveletHHLglcmImc1CTINTRA, waveletLHHglszmSizeZoneNonUniformityNormalizedCTINTRA, waveletHLHglszmSizeZoneNonUniformityCTAINTRA, waveletLLHfirstorderMeanCTAINTRA, waveletLHLngtdmBusynessCTAPERI, waveletHHHfirstorderMeanCTPERI, originalngtdmBusynessCTPERI, waveletHHHglcmIdCTAPERI, waveletLLHglszmGrayLevelNonUniformityCTPERI, waveletLLLfirstorderKurtosisCTPERI, waveletHHLglszmSmallAreaEmphasisCTAPERI.

### Models performances

**Figure 4:**
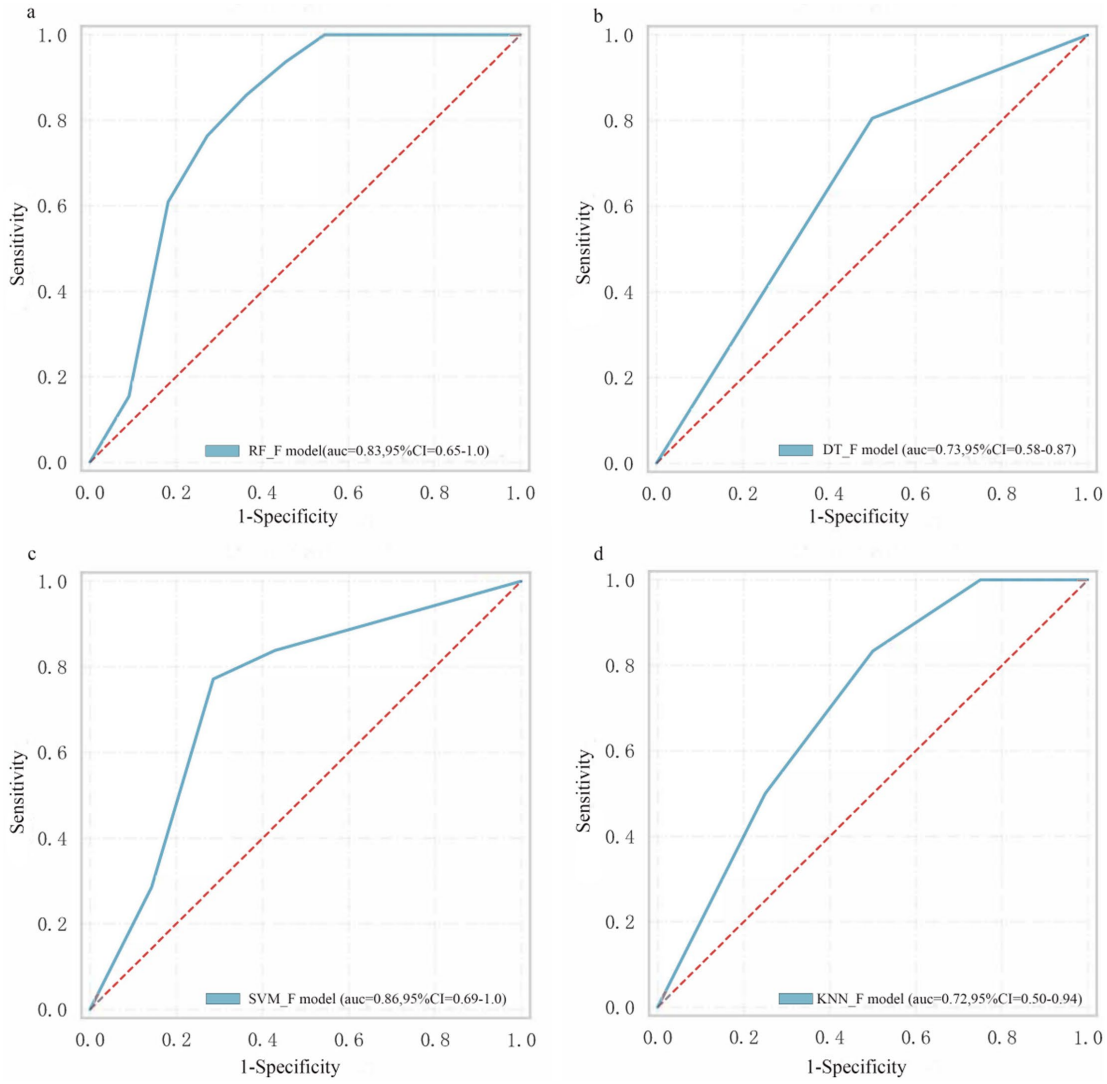
The performance of machine learning models. **(a)** RF_F model. **(b)** DT_F model. **(c)** SVM_F model. **(d)** KNN_F model.

Table 2 shows the model performance for intrathrombus, perithrombus, and fusion areas. For the intrathrombus region, the AUC values for RF-INTRA, DT-INTRA, SVM-INTRA, and KNN-INTRA are 0.78, 0.70, 0.67, and 0.69, respectively. For perithrombus models, the AUC values for RF-PERI, DT-PERI, SVM-PERI, and KNN-PERI are 0.76, 0.69, 0.73, and 0.64, respectively. Fusion models, which combined intrathrombus and perithrombus features, had AUC values for the RF-F, DT-F, SVM-F, and KNN-F models as 0.83, 0.73, 0.86, and 0.73, respectively (Figure 4). Among the models tested, only RF_INTRA, KNN_INTRA, and RF_PERI yielded LR^+^ values above 5, indicating potential clinical utility for ruling in disease. However, none of the models achieved LR^−^ values below 0.2, suggesting limited utility for ruling out disease.

**Table 2:** The performances of different machine learning models.

Model	Sensitivity	Specificity	AUC (95% CI)	LR^+^	LR^−^
RF_INTRA	0.77	1.0	0.78 (0.61–0.96)	∞	0.23
DT_INTRA	0.45	0.85	0.70 (0.55–0.86)	3.00	0.65
SVM_INTRA	0.85	0.33	0.67 (0.50–0.84)	1.27	0.45
KNN_INTRA	0.40	0.94	0.69 (0.53–0.86)	6.67	0.64
RF_PERI	0.3	0.95	0.76 (0.55–0.97)	6.00	0.74
DT_PERI	0.3	1.0	0.69 (0.51–0.95)	∞	0.70
SVM_PERI	0.62	0.62	0.73 (0.57–0.89)	1.63	0.61
KNN_PERI	0.30	0.88	0.64 (0.50–0.82)	0.94	1.03

Positive likelihood ratio (LR+), negative likelihood ratio (LR^−^).

According to Table 3, the RF_F model demonstrated the highest sensitivity (0.79) and specificity (0.71), along with the most favorable LR^+^ (2.72) and LR^−^ (0.30) values. The SVM_F model showed comparable performance with sensitivity of 0.78, specificity of 0.69, LR^+^ of 2.52, and LR^−^ of 0.32. The DT_F and KNN_F models exhibited relatively lower performance across all metrics, with DT_F having the lowest specificity (0.52) and KNN_F showing sensitivity and specificity values of 0.70 and 0.59, respectively.

**Table 3:** The performances of different machine learning models.

Model	Sensitivity	Specificity	LR^+^	LR^−^
RF_F	0.79	0.71	2.72	0.3
DT_F	0.72	0.52	1.56	0.48
SVM_F	0.78	0.69	2.52	0.32
KNN_F	0.7	0.59	1.71	0.51

Positive likelihood ratio (LR+), negative likelihood ratio (LR^−^).

### SHAP

Figure 3 illustrates the contribution of each parameter to the RF_F model, as analysed by the SHAP algorithm. Among the parameters, WaveletLHHglszmSizeZoneNonUniformityCTAINTRA emerged as the most significant variable, representing the primary RFs factor influencing the CIR, whileWaveletHHHglcmIdcCTAPERI is the most influential perithrombus RFs affecting the CIR.

## Discussion

Endovascular intervention for AIS often benefits most patients; however, some may not achieve a favorable prognosis despite successful recanalization. Therefore, accurately identifying these patients is critical. In this study, we developed and validated a radiomics-based machine learning model, which uses thrombus and peri-thrombus areas to accurately predict CIR in patients with AIS undergoing EVT treatment. We also used the SHAP algorithm to interpret the RF-F model and found that waveletLHHglszmSizeZoneNonUniformityCTAINTRA is the most important parameter for predicting CIR.

Current thrombus radiomics research primarily investigates thrombus composition (12) and its association with the number of thrombectomy attempts (15). Studies have confirmed that thrombus radiomics is related to the likelihood of successful recanalization, while the number of intervention attempts correlates with key patient prognostic factors (16, 17). In this study, nine features were extracted from intra-thrombus and seven from peri-thrombus regions, enabling the development of machine learning models that reliably predict the successful recanalization post-EVT in patients. Histopathological analysis indicates that thrombectomy often induces vascular wall injury (18), causing intimal damage, vascular wall thickening, inflammation, and blood–brain barrier disruption (19–21), contributing to a poor prognosis. To address this, we included radiomics information from a 1 mm peri-thrombus region to provide insight into the intrinsic characteristics of the vascular wall and surrounding tissues, thereby developing a predictive model that provides accurate prognosis predictions.

This study combined the mRMR and LASSO algorithms to select prognostically significant, minimal correlated variables among the model parameters, enhancing the model’s predictive performance and accuracy.

Radiomic features from both the intra-thrombus and peri-thrombus regions were extracted, enabling a comprehensive characterization of thrombus composition, vascular wall status, and perivascular inflammatory response. A hybrid machine learning model integrating both regions was developed and demonstrated superior predictive performance compared to single-region models.

Specifically, the random forest (RF) models based on intra-thrombus, peri-thrombus, and combined radiomic features all exhibited good overall predictive performance. Among them, the RF_INTRA and RF_PERI models achieved positive likelihood ratios (LR^+^) of ∞ and 6.00, respectively, indicating strong clinical utility in confirming patients at high risk of poor cerebral infarct resolution (CIR) after EVT. The KNN_INTRA model also yielded a high LR^+^ of 6.67. However, none of the models reached an LR^−^ below 0.2, suggesting limited value for ruling out poor outcomes with high confidence. These findings support the use of radiomics-based machine learning models as clinically useful tools for risk stratification, particularly when the test result is positive. The LR values provide a practical and interpretable framework for applying these models in individual clinical decision-making.

The SHAP algorithm was applied to interpret the RF_F model, further clarifying the influence of each variable on the model and ranking their importance. Wavelet LHHglszm Size Zone NonUniformity CTAINTRA was identified as the most important parameter influencing patient prognosis. This parameter reflects the uneven distribution of grayscale region sizes, with higher values indicating greater disparity, while the multiscale features introduced by the wavelet transform enable the assessment of image texture complexity and structure at different levels, revealing high heterogeneity within the thrombus.

This study had several limitations. First, the relatively small sample size limits the generalizability of the findings, necessitating validation with a large independent dataset. Second, while thrombus segmentation was performed manually, and rigorous statistical methods such as ICC analysis were applied during subsequent feature selection, some subjective bias remained.

In conclusion, this study highlights the value of radiomics in analysing thrombus and peri-thrombus features to predict CIR in AIS patients following EVT. The radiomics-based machine learning model offers a robust tool for identifying patients at risk of poor outcomes and holds promise for improving clinical decision-making. However, further study is needed to validate these findings in larger, independent cohorts and explore the broader clinical applicability of the model.

## Data Availability

The raw data supporting the conclusions of this article will be made available by the authors, without undue reservation.
